# Management of Cerebrospinal Fluid Leak following Posterior Cranial Fossa Surgery

**DOI:** 10.12669/pjms.326.9956

**Published:** 2016

**Authors:** Imran Altaf, Anjum Habib Vohra, Shahzad Shams

**Affiliations:** 1 Dr. Imran Altaf, MS. Department of Neurosurgery, Khawja Muhammad Safdar Medical College, Sialkot, Pakistan; 2 Dr. Anjum Habib Vohra, FRCS, Department of Neurosurgery, Post Graduate Medical Institute, Lahore General Hospital, Lahore, Pakistan; 3 Dr. Shahzad Shams, FRCS, FCPS, Department of Neurosurgery, Post Graduate Medical Institute, Lahore General Hospital, Lahore, Pakistan

**Keywords:** *Cerebrospinal fluid leak*, *Posterior cranial fossa surgery*, Cerebrospinal fluid lumbar drainage

## Abstract

**Objective:**

Cerebrospinal fluid leakage remains a significant cause of morbidity following posterior fossa surgery, and its treatment remains a difficult problem. The aim of the study was to propose a treatment algorithm for its management.

**Methods:**

A retrospective, single-center study was conducted on 147 patients who underwent elective posterior fossa surgery ***for a variety of diseases***. Patients with post operative CSF leakage had either been treated initially with conservative measures including re-suturing of the wound, with CSF lumbar drainage to be employed in case the CSF leakage didn’t stop, or the initial intervention was the institution of CSF lumbar drainage simultaneously with conservative measures. VP (ventriculo-peritoneal) shunt was done in patients with gross hydrocephalus on postoperative CT brain.

**Results:**

There were 25 (17%) cases of CSF leakage, including 24 incisional CSF leaks and one case of CSF otorrhea. In eight patients with incisional CSF leakage treated initially with conservative measures including re-suturing of the wound, CSF leakage stopped in only two cases. CSF lumbar drainage instituted later on in six cases with persistent leakage stopped the CSF leakage. In fourteen patients managed initially with re-suturing of the wound and concomitant CSF lumbar drainage, CSF leakage settled in all the cases. Two patients with gross hydrocephalus on post operative CT were managed successfully with VP shunt. Re-suturing of the wound with concomitant CSF lumbar drainage was found to be significantly associated (p=0.003) with the stoppage of CSF leakage, and the settlement of meningitis (p= 0.014).

**Conclusion:**

Incisional ***CSF*** leaks after posterior fossa surgery should be managed with re-suturing of the wound and concomitant ***CSF*** lumbar drainage, instead of an initial trial of conservative therapy alone.

## INTRODUCTION

Cerebrospinal fluid (CSF) leak remains a significant source of morbidity in neurosurgery, particularly after posterior fossa surgery.^1^ It is six times more likely to occur in the infratentorial procedures than in the supratentorial procedures.^2^ The incidence in posterior fossa surgery can be as high as 17%.^1^ CSF leakage poses a risk of significant morbidity and remains potentially life-threatening due to the risk of meningitis.^1^,^3^,^4^ Furthermore, the costs related to treating patients affected by this complication have been estimated to be 141% greater than that of patients without a CSF leak.^1^

Treatment of postoperative *CSF* leak following posterior fossa surgery remains a difficult and perplexing problem.^5^ Treatment options include either to start with conservative measures including re-suturing of the wound and to opt for CSF lumbar drainage in case the CSF leakage doesn’t stop,^6^,^7^ or to institute CSF lumbar drainage simultaneously with the conservative measures as the initial intervention.^8^ Surgical repair is done in case these measures fail.^9^ Studies conducted on the topic have shown conflicting results and there is no consensus on the optimal method of treatment. The purpose of our study was to retrospectively assess the efficacy of both these treatment regimens, and to propose a treatment algorithm for the management of cerebrospinal fluid leakage following posterior cranial fossa surgery.

## METHODS

The study was a retrospective, single-center study conducted in the Department of Neurosurgery, Unit-1, Lahore General Hospital from January 15, 2012 to September 15, 2015 for forty five months. Patients of both sexes and all age groups operated for posterior fossa pathologies were included in the study. Post-operative CSF leakage at any point in time was noted. Incisional *CSF* leak was diagnosed when clear fluid was observed draining through the incision, irrespective of the development of central nervous system infection. CSF rhinorrhea or otorrhea was diagnosed when clear fluid was observed draining through the nose or ear respectively.

In one third of the patients developing incisional *CSF* leakage the initial mode of treatment was the institution of conservative measures. These conservative measures included bed rest, head elevation, local wound care (such as pressure dressing) and suturing of incisional leaks. In case the CSF leak didn’t stop, CSF lumbar drainage was started. In the remaining patients developing incisional CSF leakage, re-suturing of the wound was accompanied by concomitant CSF lumbar drainage. A surgical re-exploration was to be employed if these non-operative measures failed to stop the CSF leakage. A VP shunt was done in patients having gross hydrocephalus on post-operative CT brain. Cases of otorrhea were also initially managed conservatively. Surgical re-exploration was to be done if the otorrhea didn’t settle.

The development of meningitis in any patient at any point in time was also noted. The follow up of all the patients was of two months. The collected data was entered and analyzed using SPSS version 20. Qualitative data like gender, intervention type, CSF leakage, leakage stopped and meningitis settled are presented in the form of frequency and percentages. Mean ± S.D was used for age, time of interventions and meningitis settled. Chi-square test was applied for comparison of types of interventions and leakage stopped / meningitis settled. P-value ≤ 0.05 was considered as significant.

## RESULTS

One hundred forty seven cases with posterior fossa pathologies were operated in the Department of Neurosurgery, Unit-1, Lahore General Hospital from January 15, 2012 to September 15, 2015. Of the one hundred forty seven cases of posterior fossa surgeries, retrosigmoid suboccipital posterior fossa craniectomies were carried out in seventy cases. Fifty of these suboccipital retrosigmoid craniectomies were carried out for CP Angle SOLs (space occupying lesions), and twenty were done for microvascular decompression of trigeminal neuralgia. Midline suboccipital craniectomies were done in seventy seven cases. Fifty nine of these were done for 4^th^ ventricle/cerebellar sols, twelve for foramen magnum SOLs, and six for chiari malformations. CSF leakage occurred in 7/50 (14%) cases of retrosigmoid surgeries for Cerebellopontine (CP) Angle SOLs, 12/59 (20.3%) cases of midline suboccipital craniectomies for 4^th^ ventricle/cerebellar SOLs, 4/12 (33.3%) cases of foramen magnum SOLs, and 2/6 (33.3%) cases of chiari malformations. There were no cases of CSF leakage after microvascular decompression (MVD) for trigeminal neuralgia in 20 cases.

**Fig.1:**
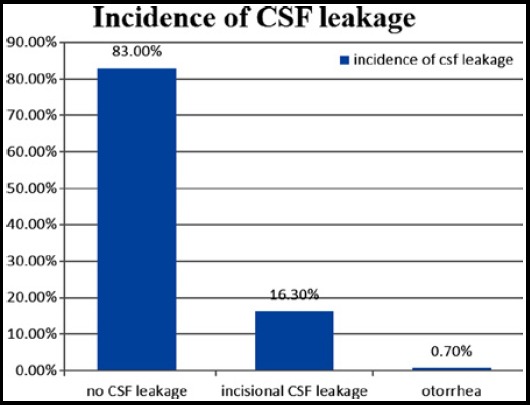
Incidence of CSF leakage.

Over all, CSF leakage occurred in twenty five (17%) cases. Out of these twenty five cases, there was incisional CSF leakage in twenty four cases, and there was a single case of otorrhea. The incidence is shown in Fig.1.

Out of the twenty four cases of incisional CSF leakage, eight patients were managed initially with conservative measures including bed rest, head elevation, local wound care (such as pressure dressing) and re-suturing of the wound. In only two (25%) cases the CSF leakage settled with these conservative measures. CSF lumbar drainage was then instituted in the remaining six (75%) patients, and that lead to the cessation of CSF leakage.

In fourteen patients the initial management was re-suturing of the wound along with concomitant CSF lumbar drainage. In all these fourteen cases the CSF leakage stopped. In two cases CSF leakage was accompanied by gross hydrocephalus on postoperative CT and a ventriculoperitoneal (VP) shunt was the intial intervention in these cases and the CSF leakage stopped. There was a single case of otorrhea that settled with conservative expectant management. The initial management was found to be significantly associated (p=<0.05) with the stoppage of CSF leakage in case of an incisional leak as shown in Table-I.

**Table-I:** Type of CSF Leakage and 1st intervention.

1st Intervention	Number of Patients	CSF Leakage Stopped	Required 2^nd^ Intervention	p-value
VP shunt	2	2	0	0.003
Re-suturing	8	2	6	
Re-suturing + CSF Lumbar drainage	14	14	0	

The stoppage of CSF leakage was significantly associated with the initial management (p=0.003). The details of age, timing of first and second intervention, and timing of developing meningitis are given in Table-II.

**Table-II:** Timing of intervention and development of meningitis

	Age	Timing of 1st intervention (days)	Timing of 2nd intervention (days)
n	147	24	6
Mean	32.40	9.12	16.00
Std. Deviation	15.24	4.20	8.22
Minimum	4.00	3.00	9.00
Maximum	69.00	22.00	27.00
Q1	20.00	7.25	9.75
Q2	35.00	9.00	12.00
Q3	43.00	11.00	26.25

Four (50%) of the eight patients with incisional CSF leakage initially managed conservatively with resuturing of the wound alone also developed meningitis. Two of these patients developed meningitis before conservative measures were instituted, and two developed meningitis after conservative measures had been instituted. Although CSF leakage settled with CSF lumbar drainage, the condition of these four patients didn’t improve.

Of the fourteen patients managed initially with re-suturing and simultaneous CSF lumbar drainage, eight (57%) developed meningitis. Of these eight patients, four (28.5%) had already developed meningitis before the CSF drainage was started. However, not only did the CSF leakage stop but the meningitis also settled in these patients, and they were discharged later on. In the other four (28.5%) patients meningitis developed after CSF lumbar drainage had been started. Two of these patients recovered and were discharged, while two patients didn’t recover from the meningitis although the CSF leakage stopped. The single patient suffering patient suffering from otorrhea didn’t develop meningitis.

The first intervention was found to be significantly associated (p= 0.014) with the settlement of meningitis in cases of incisional CSF leakage as shown in Table-III.

**Table-III:** Results of intervention

		Meningitis settled		Total	p-value

		Yes	No		
1st Intervention	Re-suturing	0	4	4	0.014
	Re-suturing + Csf Lumbar drainage	6	2	8	

Total		6	6	12	

## DISCUSSION

Cerebrospinal fluid leakage remains a significant cause of morbidity after posterior fossa surgery.^10^ When present, this complication significantly increases the risk of bacterial meningitis and often requires costly methods of treatment that include prolonged hospital stay, CSF lumbar drainage, and/or ventriculoperitoneal shunt insertion and possible surgical revisions.^11^-^13^

Although the incidence of CSF leakage after posterior fossa surgery has been quoted to be as high as 26.7%^14^, the incidence ranges between 4 and 17% in most series.^10^,^15^ The incidence of 17% in our study was thus similar to these findings. The incidence after CP Angle retrosigmoid approach in our study was 14%, which falls within the range of 10 -17.6%^16^,^17^ quoted in literature. CSF leakage has also been described as one of the most common complications following microvascular decompression (MVD) surgeries.^18^ The incidence has been reported to range from 1-12.5% after MVD for trigeminal neuralgia.^11^,^19^ However, there was no case of CSF leakage after MVD in our series, which is similar to the findings of Bayazit et al.^6^ where there was no CSF leakage after MVD.

Treating postoperative CSF leakage following posterior fossa surgery is a difficult propositon.^5^ The primary decision to be taken is to whether treat the patients conservatively with re-suturing of the wound alone, or to opt for invasive measures such as CSF lumbar drainage simultaneously with re-suturing as the initial mode of treatment. Studies conducted on the topic have shown conflicting results and no consensus exists on the optimal method of treatment.

Bayazit et al.^6^ in their study found that ten out of the thirty two patients that had CSF leakage after retrosigmoid posterior cranial fossa surgery could be managed conservatively. Twelve patients needed CSF lumbar drainage. Surgical re-exploration was performed in the remaining ten patients. They concluded that initially a conservative treatment should be instituted in the case of a CSF leak. If the conservative treatment failed the placement of a CSF lumbar drain should be considered. Patients that did not respond to lumbar drainage required surgical re-exploration. In their opinion this algorithm represented the safest and best option for the management of CSF leakage, as confirmed by the absence of recurrences or multiple revisions in their study. The exact same treatment algorithm for treating CSF leakage was proposed by Mangus et al.^7^

However, Fishman et al.^8^ in their study found conflicting results to these studies. While evaluating the efficacy of lumbar CSF drainage for the management of CSF leakage following posterior fossa surgery, Fishman et al found that CSF lumbar drainage successfully stopped the leak in eighty seven percent of the cases. There were no cases of meningitis associated with CSF lumbar drainage. They recommended that lumbar CSF drainage be started immediately after CSF leakage instead of an initial trial of conservative therapy. In the study of Allen et al.^9^, however, only half the cases of CSF leakage after suboccipital retrosigmoid surgery settled with CSF lumbar drainage.

In our study CSF leakage stopped in only two of the eight patients managed conservatively, and the remaining six patients required CSF diversion in the form of CSF lumbar drainage. Four of these eight patients developed meningitis and this meningitis didn’t settle later on. In the fourteen cases managed initially with re-suturing of the wound and concomitant CSF lumbar drainage, CSF leakage stopped in all the patients. Meningitis also settled in six of the eight patients that had developed meningitis in this group. The settlement of CSF leakage (p <0.05), and settlement of meningitis (p <0.05) were significantly associated with the first intervention. The results of our study lead to the conclusion that conservative measures such as re-suturing the wound alone are not successful in stopping CSF leakage after posterior fossa surgery. Infact, an initial trial of conservative therapy alone increases the morbidity associated with meningitis. Our findings are thus consistent with the findings of Fishman et al^8^ that had recommended that lumbar CSF drainage be started immediately after CSF leakage instead of an initial trial of conservative therapy.

### Limitations of the study

Other variables that may have influenced the risk of CSF leak, including poor wound healing (malnutrition, diabetes mellitus, concurrent glucocorticoid administration) were not tracked, possibly confounding the analysis. Also, these surgical results have been pooled by three neurosurgeons at a single institution—as a result, individual variations in operative protocol unrelated to the above discussion might also potentially confound data analysis.

## CONCLUSION

Incisional CSF leaks after posterior fossa surgery should be managed with re-suturing of the wound and concomitant CSF lumbar drainage, instead of an initial trial of conservative therapy alone.
